# High-fat diet, triglyceride glucose index, and gastrointestinal cancer: integrative insights from human and animal studies

**DOI:** 10.3389/fnut.2026.1734895

**Published:** 2026-04-01

**Authors:** Jinmei Li, Zi Dai, Yinping Cai, Ye Wu, Yalan Wu, Cantu Fang, Yao Wang

**Affiliations:** 1 School of Basic Medical Sciences, Guangzhou University of Chinese Medicine, Guangzhou, Guangdong, China; 2 State Key Laboratory of Traditional Chinese Medicine Syndrome, Guangzhou University of Chinese Medicine, Guangzhou, Guangdong, China; 3 Guangzhou University of Chinese Medicine, Guangzhou, Guangdong, China; 4 Zhongshan Hospital of Traditional Chinese Medicine Affiliated to Guangzhou University of Traditional Chinese Medicine, Zhongshan, Guangdong, China; 5 The Tenth Clinical Medical College of Guangzhou University of Traditional Chinese Medicine, Zhongshan, Guangdong, China

**Keywords:** animal studies, gastrointestinal cancer, GBD2021, high-fat diet, NHANES, TyG

## Abstract

**Background:**

High-fat diet (HFD) exposure has been associated with an increased risk of gastrointestinal (GI) cancers; however, the metabolic and inflammatory mechanisms underlying this association remain incompletely understood. Identifying clinically accessible biomarkers that capture diet-induced metabolic stress may improve early risk stratification and prevention strategies.

**Methods:**

Data were obtained from the National Health and Nutrition Examination Survey (NHANES, 2003–2020) and the Global Burden of Disease (GBD) 2021 dataset. HFD-related metabolic exposure was characterized using eight lipid-related obesity indices, including body mass index (BMI), waist-to-height ratio (WHtR), lipid accumulation product (LAP), visceral adiposity index (VAI), triglyceride–glucose index (TyG), TyG–WHtR, waist circumference (WC), and TyG–BMI. Threshold effects of TyG were evaluated, and mediation analyses were performed to assess the roles of the systemic immune-inflammation index (SII) and neutrophil-to-lymphocyte ratio (NLR). Complementary animal experiments were conducted to examine changes in TyG levels, gastrointestinal histopathology, and serum carbohydrate antigen 19–9 (CA19-9).

**Results:**

Elevated TyG levels were significantly associated with increased GI cancer risk. A nonlinear relationship was identified, with a threshold effect at TyG = 9.657; below this threshold, the association exhibited a dose-dependent pattern. Mediation analyses indicated that SII and NLR partially mediated the TyG–GI cancer association, accounting for 1.6 and 2.8% of the total effect, respectively. Stratified analyses revealed significant heterogeneity across demographic subgroups. In animal models, HFD exposure resulted in elevated TyG levels, gastrointestinal mucosal injury, and increased tumor-associated biomarkers, consistent with the development of precancerous lesions.

**Conclusion:**

The findings identify the TyG index as a practical and biologically relevant marker linking HFD-induced metabolic dysregulation to GI cancer risk, with systemic inflammation serving as a partial mediator. The integration of population-based analyses with experimental evidence underscores the potential utility of TyG in metabolic risk assessment and precision prevention strategies for GI cancers.

## 1 Introduction

Gastrointestinal (GI) cancers remain among the leading causes of cancer-related mortality worldwide and continue to pose a substantial burden on global public health (1). Despite advances in diagnostic techniques and therapeutic strategies, the overall prognosis of GI malignancies—including colorectal, gastric, hepatic, pancreatic, and esophageal cancers—remains unsatisfactory, largely due to late-stage diagnosis and limited options for effective prevention. In parallel with rapid socioeconomic development, chronic overnutrition has become increasingly prevalent, with high-fat diet (HFD) emerging as a prominent and modifiable risk factor for a wide range of metabolic disorders, including obesity, type 2 diabetes, and cardiovascular diseases (2). Accumulating evidence indicates that HFD is also closely linked to the initiation and progression of GI cancers. In 2022, colorectal cancer accounted for approximately 1.9 million new cases and over 900,000 deaths globally, ranking third in incidence and second in mortality (3). Moreover, global projections suggest that the burden of colorectal cancer will continue to rise substantially over the coming decades, reaching an estimated 3.2 million new cases and 1.6 million deaths annually by 2040 (4). Stomach cancer similarly represents a major contributor to the global cancer burden, with nearly 1 million new cases diagnosed worldwide in 2022 (3). These trends underscore the urgent need to identify metabolic and dietary factors that may contribute to GI cancers and serve as targets for early risk stratification and prevention.

Mechanistically, HFD-induced metabolic dysfunction has been proposed to be associated with GI tumorigenesis through multiple interconnected pathways, including insulin resistance, dysregulated lipid metabolism, altered adipokine secretion, chronic low-grade inflammation, and disruption of gut microbiota homeostasis (5–7). Among these mechanisms, persistent systemic inflammation is increasingly recognized as a key driver linking metabolic stress to cancer development (8). In recent years, composite hematological indices such as the systemic immune-inflammation index (SII) and the neutrophil-to-lymphocyte ratio (NLR) have gained attention as convenient and clinically accessible markers of low-grade inflammation, reflecting the integrated activity of innate and adaptive immune responses. These indices have been associated with obesity-related metabolic abnormalities as well as increased cardiometabolic and GI risk (9, 10).

Although the epidemiological association between HFD and GI cancers is well established, clinically applicable biomarkers that capture the metabolic–inflammatory axis underlying this relationship remain limited. The triglyceride glucose (TyG) index, a validated surrogate marker of insulin resistance, has recently attracted increasing interest due to its close association with systemic inflammation and metabolic stress (9). Emerging evidence suggests that elevated TyG levels are positively correlated with inflammatory indices such as SII and NLR, supporting a mechanistic link between insulin resistance, chronic inflammation, and cancer-related risk (10–13). Furthermore, higher TyG index values have been associated with an increased risk of GI cancers, highlighting the potential role of metabolic dysregulation in the development of GI cancers (14).

**Figure 1:**
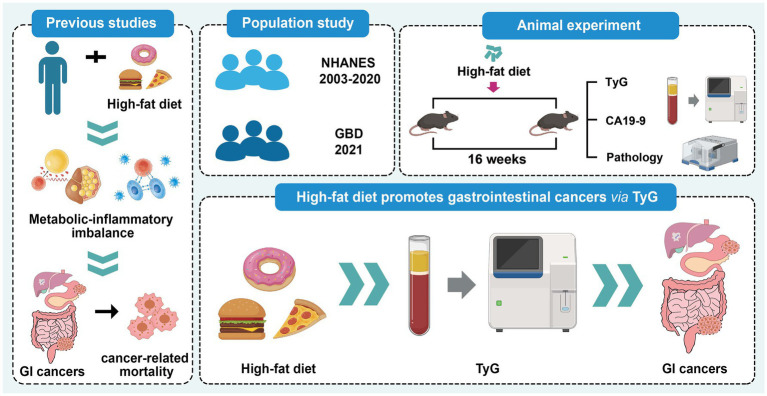
Diagram of the study.

The present study aimed to investigate the association between high-fat dietary intake and the risk of GI cancers, with particular emphasis on the potential mediating role of the TyG index. By combining large-scale epidemiological analyses with complementary animal experiments, this work provides a more comprehensive assessment of the metabolic and inflammatory pathways linking HFD to the development of GI cancers. The overall study design is summarized in Figure 1.

## 2 Materials and methods

### 2.1 Study population

The GBD 2021 results, available at [Global Burden of Disease Results] (https://vizhub.healthdata.org/gbd-results), are managed by the Institute for Health Metrics and Evaluation (IHME) at the University of Washington, USA. In this study, we extracted age-standardized death rates (ASDRs) for five major gastrointestinal cancers across 204 countries and territories to assess the global mortality burden. According to GBD classifications, the codes for these five gastrointestinal cancer types include: C18–C21.9, D01.0–D01.3, D12–D12.9, D37.3–D37.5 for colorectal cancer (CRC); C22–C22.9, D13.4 for liver cancer; C16–C16.9, D00.2, D13.1, D37.1 for stomach cancer; C15–C15.9, D00.1, D13.0 for esophageal cancer; and C25–C25.9, D13.6–D13.7 for pancreatic cancer.

Data were obtained from the National Health and Nutrition Examination Survey (NHANES), a continuous, nationally representative research program designed to assess the health and nutritional status of the U.S. civilian, noninstitutionalized population using a stratified, multistage probability sampling design. NHANES is conducted in 2-year cycles and integrates data from multiple components, including demographic characteristics, dietary intake, physical examinations, laboratory measurements, and health-related questionnaires. Detailed information regarding survey design, data collection procedures, and analytic guidelines is publicly available on the official NHANES website.^1^

**Figure 2:**
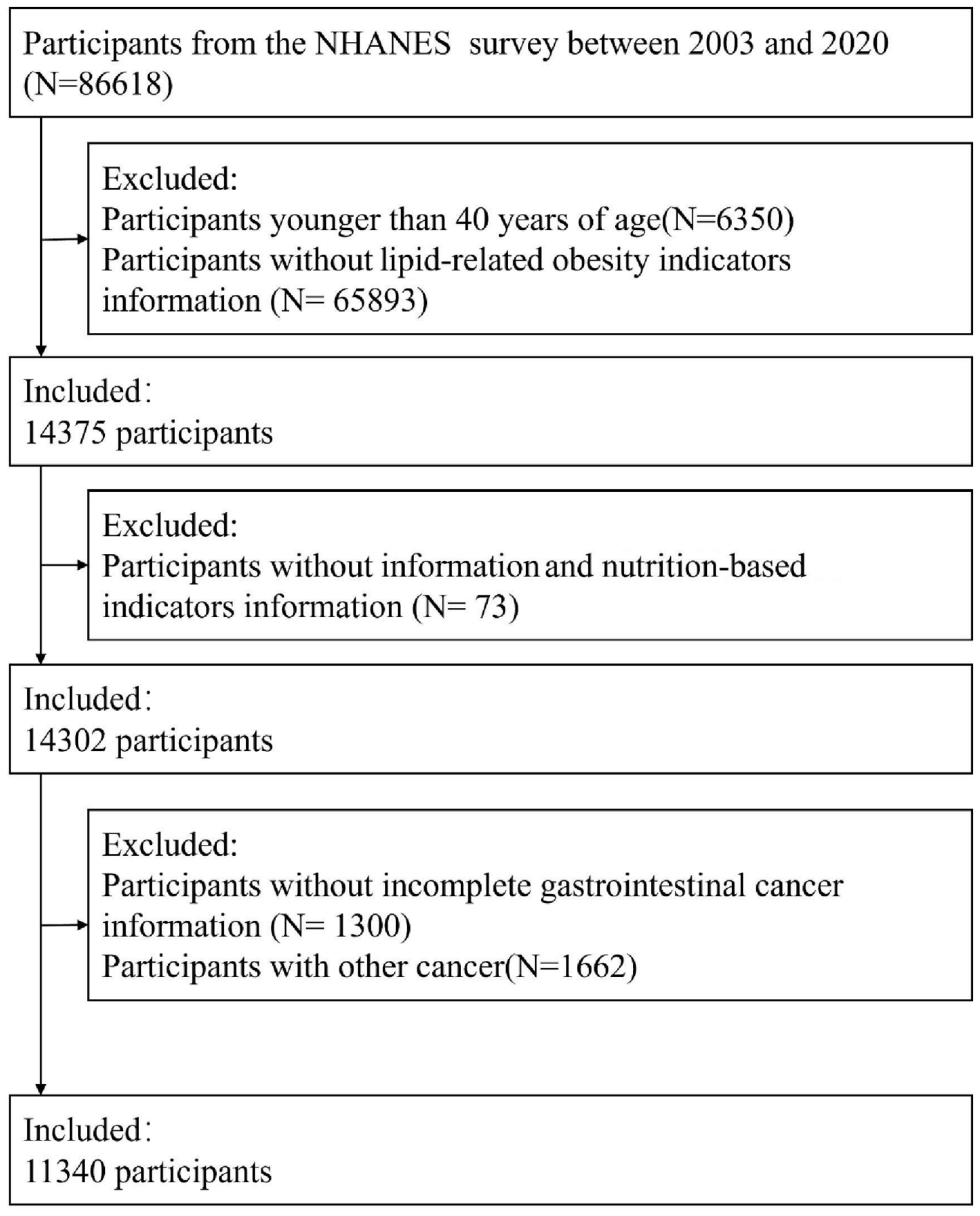
Screening flow chart of the research population.

A total of 86,618 participants from NHANES cycles conducted between 2003 and 2020 were initially considered. Participants were excluded sequentially according to predefined criteria to ensure data completeness and analytical consistency. Individuals younger than 40 years (*n* = 6,350) and those with missing data on lipid-related obesity indicators (*n* = 65,893) were excluded. Subsequently, 73 participants lacking information on inflammatory or nutrition-related biomarkers were removed. An additional 1,300 participants with incomplete GI cancers data and 1,662 individuals diagnosed with non-GI cancers were excluded. After these exclusions, the final analytical sample consisted of 11,340 eligible participants. The participant selection process is illustrated in Figure 2.

### 2.2 Assessment of lipid-related obesity indicators

A total of eight lipid-related obesity indicators were included in this study, and their specific calculation methods, measurement standards, and corresponding formulas are summarized in Table 1. Body Mass Index (BMI) and Waist Circumference (WC) serve as general indicators of overall and abdominal obesity, respectively. Visceral Adiposity Index (VAI) and Lipid Accumulation Product (LAP) reflect visceral adiposity and lipid accumulation by integrating anthropometric and lipid parameters. Waist-to-Height Ratio (WHtR) evaluates abdominal obesity relative to stature. The Triglyceride-Glucose (TyG) index and its composite derivatives (TyG-BMI and TyG-WHtR) capture the interplay between lipid metabolism, glucose metabolism, and adiposity, and have been proposed as surrogate markers of insulin resistance and metabolic dysfunction associated with chronic high dietary fat intake. The TyG index was calculated as ln(fasting triglycerides [mg/dL] × fasting blood glucose [mg/dL]/2), whereby the natural log transformation is incorporated into the index formula itself to normalize the skewed distribution of the product term. These indicators collectively represent a spectrum of metabolic disturbances that may underlie gastrointestinal carcinogenesis.

**Table 1:** Calculation method of eight lipid-related obesity indicators.

Lipid-related obesity indicators	Formula number	Calculation formula	References
Body mass index (BMI)	Formula 1	BMI = Weight (kg)/[Height (m)]^2^	(59)
Waist circumference	–	Measured as the abdominal girth at the umbilical level using a measuring tap	–
Visceral adiposity index (Male)	Formula 2	VAI = [WC/(39.68 + 1.88 × BMI)] × (TG/1.03) × (1.31/HDL-C)	(60)
Visceral adiposity index (Female)	Formula 3	VAI = [WC/(36.58 + 1.89 × BMI)] × (TG/0.81) × (1.52/HDL-C)	(60)
Lipid accumulation product (Male)	Formula 4	LAP *=* (WC − 65 cm) × TG concentration	(61)
Lipid accumulation product (Female)	Formula 5	LAP *=* (WC − 58 cm) × TG concentration	(61)
Waist-to-height ratio	Formula 6	WHtR = Waist Circumference (WC)/Height	–
Triglyceride glucose index	Formula 7	TyG = Natural logarithm of [fasting triglyceride (mg/dL) × fasting glucose (mg/dL)/2]	(62)
Triglyceride glucose index–body mass index	Formula 8	TyG-BMI = TyG Index × BMI	(63)
Triglyceride glucose index–waist-to-height ratio	Formula 9	TyG-WHtR = TyG Index × WHtR	(63)

### 2.3 Inflammation and nutrition-based indicators

To investigate the impact of systemic inflammatory mediators on gastrointestinal neoplastic transformation and tumor evolution, a series of inflammation-related biomarkers were analyzed. These included eight common inflammation- and nutrition-related biomarkers, namely the monocyte-to-albumin ratio (MAR), prognostic nutritional index (PNI), SII, red cell distribution width-to-albumin ratio (RAR), platelet-to-lymphocyte ratio (PLR), NLR, neutrophil-to-albumin ratio (NAR), and lymphocyte-to-monocyte ratio (LMR). These biomarkers comprehensively reflect the balance between systemic inflammation, immune function and nutritional status, which are closely related to tumor progression. Detailed information regarding their calculation approaches is compiled in Table 2.

**Table 2:** Calculation method of eight inflammation-related biomarkers.

Inflammation-related biomarkers	Formula number	Calculation formula	References
Systemic immune-inflammation index	Formula 10	Platelet count × Neutrophil count/Lymphocyte count	(52)
Neutrophil-to-albumin ratio	Formula 11	Neutrophil count/Serum albumin concentration	(64)
Prognostic nutritional index	Formula 12	Serum albumin + 5 × Absolute peripheral blood lymphocyte count	(65)
Monocyte-to-albumin ratio	Formula 13	Monocyte count/Serum albumin	(66)
Red cell distribution width-to-albumin ratio	Formula 14	Red cell distribution width (RDW)/Albumin	(67)
Neutrophil-to-lymphocyte ratio	Formula 15	Neutrophil count/Lymphocyte count	(68)
Platelet-to-lymphocyte ratio	Formula 16	Platelet count/Lymphocyte count	(68)
Lymphocyte-to-monocyte ratio	Formula 17	Lymphocyte count/Monocyte count	(68)

### 2.4 Assessment of GI cancers

GI cancers were identified based on participants’ self-reported responses to cancer-related items included in the NHANES cancer questionnaire (15). Participants who reported a diagnosis of GI cancer were classified as GI cancer cases, whereas those reporting other cancer types or no cancer diagnosis were classified as non-GI cancer cases. Six GI cancer types were included as outcome variables: pancreatic cancer, esophageal cancer, liver cancer, stomach cancer, colon cancer, and rectal cancer (16). Detailed questionnaire items, coding procedures, and classification criteria are described in the Supplementary Figure 1.

### 2.5 Covariates

The included covariates were age, sex, race, family poverty-income ratio (PIR), education status, and marital status. These variables have been confirmed by previous evidence to significantly influence the obesity-inflammation-cancer pathway (14). To ensure the robustness of the analysis, we supplemented E-value analysis to quantify the potential impact of unmeasured confounding factors on the study results, thereby further enhancing the reliability of association inferences (17).

### 2.6 Animal experiment

Specific pathogen-free male C57BL/6J mice (6 weeks of age) were used for *in vivo* experiments. Animals were randomly assigned to either a normal control (NC) group fed a standard chow diet or a high-fat (HF) group fed a high-fat diet (D12492, Research Diets) for 16 weeks, with 60% of total caloric intake derived from fat (18). Each group consisted of six mice. Animals were housed in standard polycarbonate cages (290 × 178 × 160 mm), with no more than three mice per cage, and maintained under specific pathogen-free (SPF) conditions at a constant temperature of 22 °C with a 12-h light–dark cycle. Sterilized chow and autoclaved drinking water were provided ad libitum throughout the experimental period.

At the end of the entire dietary intervention, mice were euthanized by intraperitoneal injection of an overdose of sodium pentobarbital (150 mg/kg), which was followed by cervical dislocation (19). Tissue samples from the stomach, colon, pancreas, and liver were collected and processed for histopathological evaluation using hematoxylin and eosin (H&E) staining. Serum carbohydrate antigen 19–9 (CA19-9) levels were quantified using enzyme-linked immunosorbent assay (ELISA). In addition, serum triglycerides (TG), total cholesterol (TC), fasting blood glucose (FBG), and low-density lipoprotein cholesterol (LDL-C) were measured using an automated biochemical analyzer (Mindray).

### 2.7 Statistical analysis

Given the complex sampling design of NHANES, weighted samples were applied, and SAS survey procedures were used to ensure nationally representative estimates. All statistical analyses incorporated NHANES sampling weights and complex survey design parameters. Weighted means (standard errors) summarized continuous variables, while categorical variables were reported as proportion estimates incorporating sampling weights. Group comparisons employed survey-weighted linear regression for continuous outcomes and survey-weighted χ^2^ tests for categorical variables.

Weighted multiple logistic regression models were applied to evaluate the relationship between HFD and GI cancers. Model 1 was unadjusted; Model 2 adjusted for age, sex, and race; and Model 3 further adjusted for educational attainment, family PIR, and marital status. Nonlinear associations of lipid-related obesity indicators with GI cancer risk were evaluated using two-piecewise linear regression model, with adjustment for demographic covariates including age, sex, race, education level, and marital status. The breakpoint was determined automatically using a maximum likelihood approach *via* a recursive algorithm (20). Briefly, 19 candidate values were first evaluated at 5% increments from the 5th to the 95th percentile of the TyG distribution, and the percentile yielding the highest likelihood value was recorded as p1; the search range was then narrowed to the interval between the (p1–4%) and (p1 + 4%) percentiles. A recursive procedure was subsequently applied within this range, iteratively comparing likelihood values at the Q1, Q2, and Q3 positions and eliminating 50% of candidate values at each iteration, until the optimal breakpoint k that maximized the model likelihood was identified. The 95% confidence interval of the breakpoint was determined using a Bootstrap resampling approach, whereby 1,000 datasets of identical sample size were drawn with replacement from the original data, threshold analysis was performed on each resampled dataset to obtain ki, and the 2.5th and 97.5th percentiles of the 1,000 ki values were taken as the 95% confidence interval. The existence of a threshold effect was confirmed by a likelihood ratio test (LRT) comparing the linear model against the two-piecewise regression model. All analyses were implemented using the glm() function from the stats package in R software. To test the hypothesized mediating effects, this study employed the bootstrapping method with 1,000 bias-corrected resamples to conduct mediation analysis (21). Mediation analysis was performed to evaluate whether inflammation-related biomarkers mediated the association between lipid-related obesity indicators and GI cancer risk. The total effect (TE) represented the overall association between obesity indicators and GI cancers. The indirect effect (IE) captured the mediation via inflammatory markers, while the direct effect (DE) reflected the association after accounting for these mediators. And finally, the proportion of the mediator effect, showing how much of the total effect is due to the mediator, has to be positive (22). Subgroup analyses were further performed by sex, and ethnicity.

## 3 Results

### 3.1 Global gastrointestinal cancers burden

**Figure 3:**
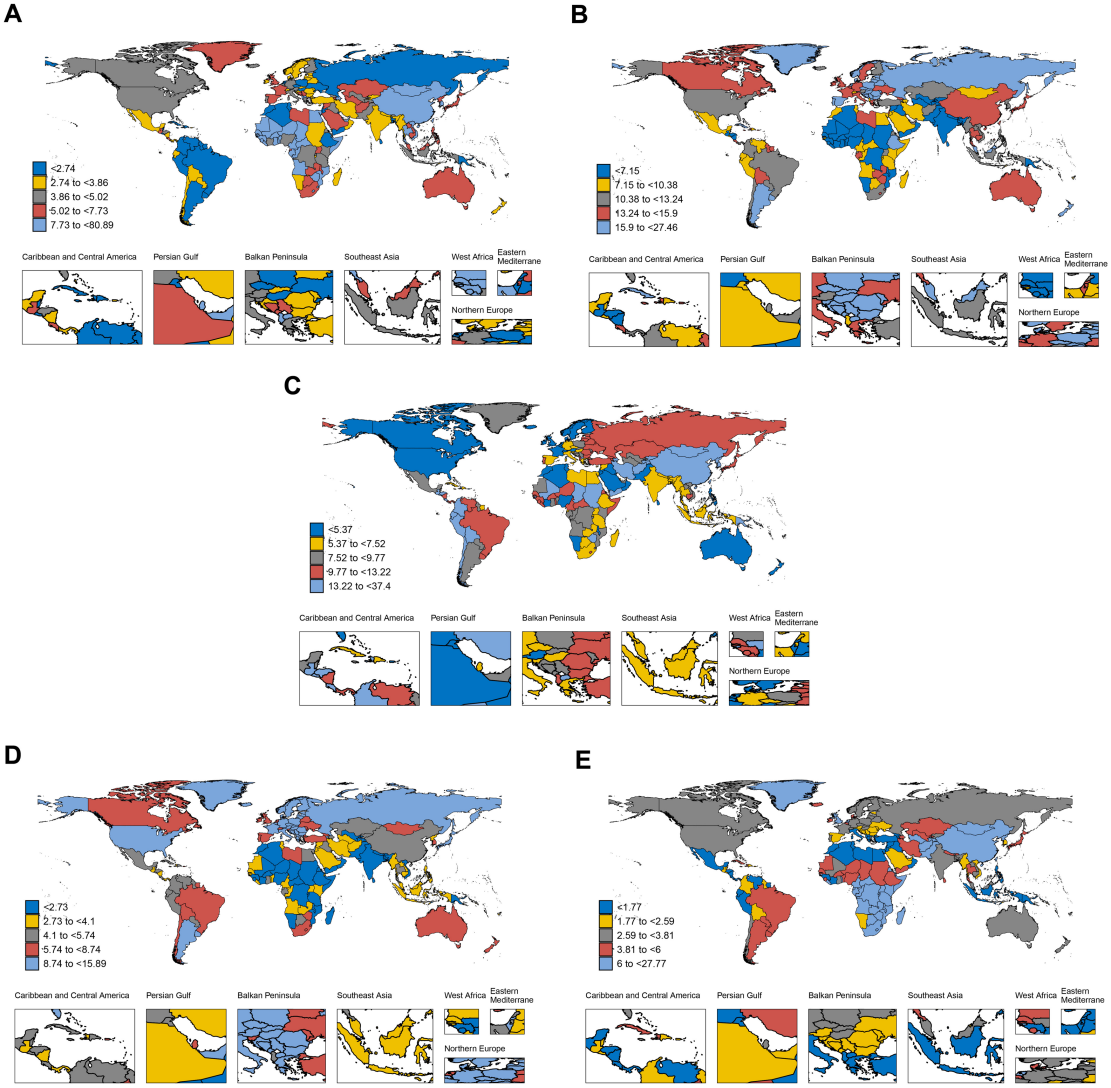
Global distribution of age-standardized death rates (ASDRs) for gastrointestinal cancers in 2021. **(A)** Liver cancer. **(B)** Colorectal cancer. **(C)** Stomach cancer. **(D)** Pancreatic cancer. **(E)** Esophageal cancer.

In 2021, substantial disparities in age-standardized death rates (ASDRs) for gastrointestinal cancers were observed across 204 countries and territories (Figures 3A–E, Supplementary Table 1). For liver cancer, Mongolia exhibited the highest ASDR at 80.895(95% uncertainty interval [UI]: 62.082–102.562), whereas Morocco reported the lowest at 0.539 (95%UI: 0.383–0.683) (Figure 3A). Marked heterogeneity was also observed for colon and rectum cancer (CRC), with Uruguay showing the highest ASDR at 27.461 (95%UI: 24.253–30.906) and Gambia the lowest at 3.047 (95%UI: 2.340–3.925) (Figure 3B). For stomach cancer, Mongolia demonstrated the highest ASDR at 37.4 (95%UI: 29.358-45.86), while Morocco again reported the lowest at 2.511 (95%UI: 1.781–3.078) (Figure 3C). For pancreatic cancer, Greenland had the highest ASDR at 15.890 (95%UI: 12.859–19.298), whereas Mozambique had the lowest at 0.903 (95%UI: 0.676–1.171) (Figure 3D). Esophageal cancer showed pronounced geographic variation, with Malawi recording the highest ASDR at 27.774 (95%UI: 22.45-34.722) and Tunisia the lowest at 0.687 (95%UI: 0.475–0.955) (Figure 3E). These findings highlight considerable global heterogeneity in the mortality burden of GI cancers.

### 3.2 Participants characteristics

After application of inclusion and exclusion criteria, a total of 11,340 eligible participants were included in the final analysis, comprising 133 individuals with incident GI cancers and 11,207 non-cancer controls. Baseline characteristics are summarized in Table 3. Five types of GI cancers were represented, including liver, stomach, esophageal, pancreatic, and colorectal cancers.

**Table 3:** Baseline.

Characteristics	Without gastrointestinal cancer	With gastrointestinal cancer	*p* value
Demographic characteristics			
Age	56.316 ± 0.168	68.164 ± 1.503	<0.001
Family PIR	3.133 ± 0.037	2.691 ± 0.180	0.012
Gender, (%)			0.179
Female	52.026	59.638	
Male	47.974	40.362	
Race/ethnicity, (%)			0.124
Mexican American	6.694	5.084	
Non-Hispanic	80.857	87.327	
Multiracial	12.449	7.589	
Education status, (%)			0.346
Below high school	17.230	21.988	
High school	41.620	44.261	
Above high school	41.116	33.750	
Unknown	0.035	0.000	
Married status			0.007
Married	55.633	36.221	
Non-married	24.424	34.995	
Unknown	19.943	28.784	
Lipid-related obesity indicators			
BMI	29.364 ± 0.107	28.607 ± 0.638	0.2396
WC	101.188 ± 0.264	101.129 ± 1.970	0.9764
VAI	1.905 ± 0.025	2.252 ± 0.174	0.0496
LAP	58.298 ± 0.781	65.295 ± 5.448	0.1994
WHTR	0.602 ± 0.002	0.610 ± 0.010	0.486
TYG	8.657 ± 0.010	8.818 ± 0.056	0.0049
TYGBMI	255.34 ± 1.112	253.307 ± 6.755	0.765
TYGWHTR	5.234 ± 0.018	5.395 ± 0.116	0.1713
Inflammation and nutrition-based indicators			
SII	550.37 ± 5.320	634.05 ± 41.569	0.0463
NAR	0.096 ± 0.001	0.100 ± 0.006	0.4815
PNI	51.502 ± 0.069	50.451 ± 0.534	0.051
MAR	0.013 ± 0.000	0.015 ± 0.001	0.0523
RAR	0.319 ± 0.001	0.341 ± 0.007	0.0013
NLR	2.234 ± 0.019	2.511 ± 0.138	0.0494
PLR	138.188 ± 0.789	157.726 ± 8.901	0.0303
LMR	3.838 ± 0.023	3.527 ± 0.162	0.0589

Family PIR, Poverty Income Ratio; BMI, Body Mass Index; WC, Waist circumference; VAI, Visceral Adiposity Index; LAP, Lipid Accumulation Product; WHTR, waist-height ratio; TYG, Triglyceride Glucose Index; SII, systemic immunity-inflammation index; NAR, neutrophil-albumin ratio; PNI, Prognostic nutritional index; MAR, Monocyte-albumin ratio; RAR, Red cell distribution width-albumin ratio; NLR, Neutrophil-to-lymphocyte ratio; PLR, Platelet-to-lymphocyte ratio; LMR, Lymphocyte-to-monocyte ratio.

Compared with participants without GI cancers, those diagnosed with GI cancers were significantly older, had lower socioeconomic status as indicated by family poverty–income ratio (PIR), and were more likely to be unmarried. With respect to lipid-related obesity indicators, significantly higher levels of the triglyceride–glucose (TyG) index (*p =* 0.0049) and visceral adiposity index (VAI) (*p =* 0.0496) were observed among GI cancer cases. Regarding inflammation- and nutrition-related biomarkers, levels of the systemic immune-inflammation index (SII) (*p =* 0.0463), red cell distribution width–to–albumin ratio (RAR) (*p =* 0.0013), neutrophil-to-lymphocyte ratio (NLR) (*p =* 0.0494), and platelet-to-lymphocyte ratio (PLR) (*p =* 0.0303) were significantly elevated in participants with GI cancers.

### 3.3 Association between GI cancers and lipid-related obesity indicators

Associations between lipid-related obesity indicators and GI cancer risk are presented in Table 4. The TyG index was significantly associated with increased GI cancer risk. In the unadjusted model (Model 1), each one-unit increase in TyG was associated with a higher odds of GI cancers (odds ratio [OR] = 1.533, 95% confidence interval [CI]: 1.151–2.042, *p =* 0.0041). This association remained statistically significant after adjustment for age, sex, and race (Model 2: OR = 1.476, 95% CI: 1.073–2.031, *p =* 0.0183). Further adjustment for educational attainment, family PIR, and marital status in Model 3 yielded a comparable estimate (OR = 1.494, 95% CI: 1.115–2.000, *p =* 0.0081), indicating a robust association. E-value analysis yielded a value of 2.35, suggesting that an unmeasured confounder would need to be associated with both the TyG index and GI cancer risk by a risk ratio of at least 2.35 to fully explain the observed association (Supplementary Figure 2), supporting the robustness of the findings to unmeasured confounding.

**Table 4:** Associations between lipid-related obesity indicators and gastrointestinal cancer.

Gastrointestinal cancer	Model 1	Model 2	Model 3
	OR (95%CI) *p* value	OR (95%CI) *p* value	OR (95%CI) *p* value
TyG	1.533 (1.151, 2.042) 0.0041	1.476 (1.073, 2.031) 0.0183	1.494 (1.115, 2.000) 0.0081
TyG quartile			
Q1	Ref.	Ref.	Ref.
Q2	1.891 (0.755, 4.735) 0.1761	1.649 (0.627, 4.335) 0.3124	1.724 (0.626, 4.747) 0.2943
Q3	1.097 (0.542, 2.222) 0.7973	0.909 (0.432, 1.910) 0.8006	0.966 (0.458, 2.038) 0.9288
Q4	2.974 (1.582, 5.592) 0.0009	2.505 (1.287, 4.874) 0.0078	2.650 (1.357, 5.175) 0.0051
*P* for trend	0.0170	0.0470	0.0251
VAI	1.135 (1.026, 1.256) 0.0149	1.144 (1.029, 1.273) 0.0142	1.154 (1.046, 1.272) 0.0050
VAI quartile			
Q1	Ref.	Ref.	Ref.
Q2	1.295 (0.558, 3.004) 0.5489	1.226 (0.522, 2.878) 0.6403	1.273 (0.518, 3.130) 0.5995
Q3	0.883 (0.480, 1.622) 0.6881	0.801 (0.434, 1.481) 0.4806	0.833 (0.449, 1.545) 0.5625
Q4	2.061 (1.182, 3.595) 0.0120	2.009 (1.135, 3.559) 0.0182	2.115 (1.204, 3.715) 0.0103
*P* for trend	0.0669	0.0902	0.0553

Model 1: no covariates were adjusted. Model 2: Age, sex, and race were adjusted. Model 3: Age, sex, race, family PIR, education status, married status were adjusted.

Ref, reference; CI, confidence interval; OR, odds ratio.

When TyG was categorized into quartiles, participants in the highest quartile (Q4) exhibited significantly higher GI cancer risk compared with those in the lowest quartile (Q1). The association persisted across all models: Model 1 (OR = 2.974, 95% CI: 1.582–5.592, *p =* 0.0009), Model 2 (OR = 2.505, 95% CI: 1.287–4.874, *p =* 0.0078), and Model 3 (OR = 2.650, 95% CI: 1.357–5.175, *p =* 0.0051). Trend tests across quartiles were statistically significant, indicating a dose-dependent relationship.

Similarly, VAI was also positively associated with GI cancers. In Model 1, the OR was 1.135 (95% CI: 1.026–1.256, *p =* 0.0149), in Model 2, the OR was 1.144 (95% CI: 1.029–1.273, *p =* 0.0142), and in Model 3, the OR was 1.154 (95% CI: 1.046–1.272, *p =* 0.0050), indicating a consistent association across models. When analyzed by quartiles, Q4 of VAI was significantly associated with higher GI cancers risk: Model 1 (OR = 2.061, 95% CI: 1.182–3.595, *p =* 0.0120), Model 2 (OR = 2.009, 95% CI: 1.135–3.559, *p =* 0.0182), and Model 3 (OR = 2.115, 95% CI: 1.204–3.715, *p =* 0.0103). However, the trend tests across VAI quartiles did not reach statistical significance, suggesting no clear dose–response pattern.

### 3.4 Subgroup analysis

Stratified analyses by gender and racial groups (Supplementary Table 2) revealed differential associations between lipid-related obesity indicators and GI cancers. Notably, the positive associations were more prominent in female participants (OR = 2.148, 95% CI: 1.554–2.969, *p <* 0.001) and in Non-Hispanic individuals (OR = 1.594, 95% CI: 1.159–2.192, *p =* 0.005).

### 3.5 Nonlinearity analysis of TyG

Nonlinear associations between TyG and GI cancer risk were evaluated using a two-piecewise linear regression model (Table 5; Supplementary Figure 3). Although the conventional linear regression model did not show a significant association (*p =* 0.103 > 0.05), the likelihood ratio test indicated that the two-piecewise model provided a significantly better fit (*p =* 0.007).

**Table 5:** The results of the two-piecewise linear regression model.

TyG	GI cancers	
	OR (95%CI)	*p* value
Fitting by linear regression model	1.271 (0.953, 1.694)	0.103
Fitting by the two-piecewise linear regression model		
Inflection point	9.657	
<9.657	1.603 (1.135, 2.264)	0.007
>9.657	0.036 (0.001, 1.324)	0.071
Log-likelihood ratio test		0.007

All adjusted for age, sex, race, Family PIR, education status, married status.

An inflection point was identified at a TyG value of 9.657. Below this threshold, a significant positive association was observed (OR = 1.603, 95% CI: 1.135–2.264; *p =* 0.007), indicating a 60.3% increase in GI cancer risk per unit increase in TyG. Above the inflection point, no significant association was detected, suggesting a plateau effect at higher TyG levels.

### 3.6 Mediating role of inflammation related indicators

Mediation analyses were conducted to assess whether inflammation-related biomarkers mediated the association between TyG and GI cancer risk (Table 6). SII was identified as a significant mediator, accounting for 1.60% of the total association (indirect effect = 0.000040, 95% CI: 0.000014–0.000175). NLR also demonstrated a significant mediating effect, contributing 2.80% of the association (indirect effect = 0.000072, 95% CI: 0.000003–0.000161). These findings suggest that systemic inflammation partially mediates the relationship between insulin resistance and GI cancer risk.

**Table 6:** Analysis of the mediation by inflammation-related indicators of the associations of triglyceride glucose and GI cancers.

	Mediation effect (95%CI), *p* value			
	Total effect	Indirect effect	Direct effect	Mediation
SII	0.002436 (0.000061, 0.004708) 0.044	0.000040 (0.000014, 0.000175) 0.002	0.002396 (0.000016, 0.004669) 0.048	1.6%
RAR	0.002477 (0.000143, 0.004662) 0.038	−0.000370 (−0.000545, −0.000198) <0.001	0.002847 (0.000464, 0.005113) 0.022	−14.9%
NLR	0.002500 (0.000085, 0.004725) 0.036	0.000072 (0.000003, 0.000161) 0.042	0.002428 (0.000052, 0.004698) 0.046	2.8%
PLR	0.002527 (0.000136, 0.004756) 0.038	**−**0.000667 (−0.000953, −0.000341) <0.001	0.003194 (0.000697, 0.005522) 0.010	−26.4%

### 3.7 Animal experiment

**Figure 4:**
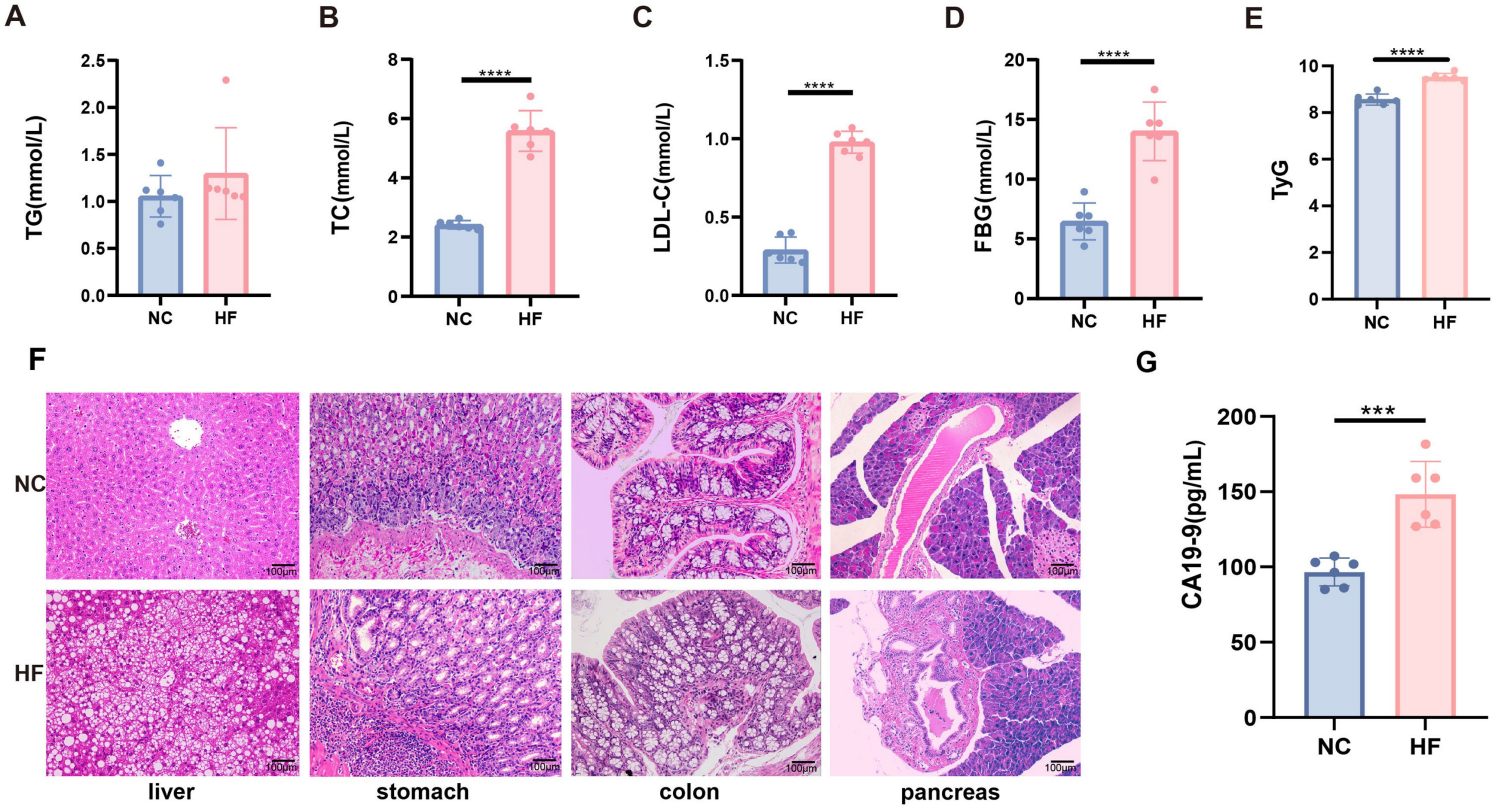
Biochemical indicators and histopathology in mice treated with different diet. **(A)** TG. **(B)** TC. **(C)** LDL-C. **(D)** FBG. **(E)** TyG. **(F)** hematoxylin and eosin (H&E)-stained sections of liver, colon, stomach, and pancreas tissues from NC and HF mice. Images were captured at ×200 magnification. Scale bar = 100 μm. G: Serum carbohydrate antigen 19-9 (CA19-9) levels. Significance levels are indicated as ^*^p < 0.05, ^**^p < 0.01, ^***^p < 0.001, ^****^p < 0.0001. TC, total cholesterol; TG, triglycerides; LDL-C, low-density lipoprotein cholesterol; FBG, fasting blood glucose; TyG, triglyceride glucose index.

Given the inherent constraints of observational designs in causal inference, this study’s human data were supplemented by controlled animal experiments to further investigate obesity-GI cancer link. High-fat diet administration resulted in significantly increased body mass compared to the NC group. Biochemical analysis revealed significantly increased levels of TC, TG, FBG, and LDL-C in the HF group relative to the NC group (Figures 4A–D). Moreover, TyG levels were markedly increased in the HF group (Figure 4E).

Histopathological examination revealed pronounced tissue alterations in HF-fed mice (Figure 4F). Liver sections demonstrated disrupted lobular architecture, hepatocellular atypia, and prominent cytoplasmic vacuolation. Colonic tissues exhibited reduced mucus secretion, crypt atrophy, glandular disorganization, and inflammatory cell infiltration. Gastric tissues showed inflammatory infiltration and features consistent with intestinal metaplasia and structural dysplasia. Pancreatic tissues from the HF group exhibited significant acinar-to-ductal metaplasia, within which dysplastic foci characterized by nuclear enlargement, hyperchromasia, and loss of polarity were identified, consistent with a diagnosis of PanIN-2 grade dysplasia. Furthermore, serum levels of the gastrointestinal tumor marker carbohydrate antigen 19–9 (CA19-9) were significantly elevated in HF-fed mice compared with controls (*p <* 0.001, Figure 4G), supporting the presence of tumorigenic alterations.

## 4 Discussion

In this study, population-based analyses and experimental validation were combined to examine the relationship between HFD related metabolic dysfunction and GI cancer risk. The results consistently indicate that the TyG index is strongly associated with GI cancer occurrence, exhibits a nonlinear threshold pattern, and is partially mediated by systemic inflammatory activity. Importantly, findings from the animal experiments provide biological support for the epidemiological observations, strengthening the plausibility of a potential mechanistic link between metabolic stress induced by HFD exposure and early gastrointestinal tumorigenesis.

Chronic exposure to high-fat diet has long been linked to GI carcinogenesis through multiple interrelated mechanisms, including insulin resistance, lipid dysregulation, endocrine imbalance, and persistent low-grade inflammation (5–7, 23). To comprehensively characterize these metabolic alterations, the present study evaluated eight lipid-related obesity indicators encompassing anthropometric measures, lipid accumulation indices, and insulin resistance–related composite markers. Among these indicators, TyG and VAI were significantly associated with GI cancer risk; however, TyG demonstrated greater robustness across adjusted models, clearer dose–response characteristics, and stronger biological interpretability. VAI reflects visceral fat accumulation and lipid dysfunction and has been associated with cancer incidence in prospective cohorts (24, 25). Nevertheless, increasing evidence suggests that the potential oncogenic effects of visceral adiposity are largely associated with insulin resistance–related mechanisms rather than adiposity per se (26–29). Although the hyperinsulinemic–euglycemic clamp remains the reference method for assessing insulin resistance, its complexity limits feasibility in large epidemiological studies. In contrast, the TyG index, derived from fasting triglyceride and glucose levels, provides a simple and validated surrogate marker of insulin resistance across diverse populations (29, 30). Consistent with prior reports linking elevated TyG levels to colorectal and gastric cancer risk (29–31), the present findings extend this observed association to overall GI cancers. A notable feature of the current analysis is the identification of a nonlinear relationship between TyG and GI cancer risk, with an inflection point at 9.657. Below this threshold, increasing TyG levels were associated with a progressively higher cancer risk, whereas no further increase was observed above this point, suggesting a possible saturation or plateau effect. Similar threshold phenomena have been reported in studies of TyG and gastric carcinogenesis (32), supporting the biological plausibility of this pattern. This finding suggests that metabolic dysregulation may be most strongly associated with carcinogenic risk within a specific range, which could have implications for early risk stratification and intervention.

From a biological perspective, insulin resistance–associated hyperinsulinemia has been proposed as a key contributor to malignant transformation. Elevated insulin and insulin-like growth factor-1 (IGF-1) signaling has been associated with cellular proliferation, inhibition of apoptosis, and enhanced angiogenesis through potential activation of the PI3K/Akt and MAPK pathways in colorectal, pancreatic, and hepatic cancers (33–39). Insulin may also cross-activate IGF-1 receptors, potentially amplifying oncogenic signaling cascades that may be involved in tumor initiation and progression (33, 34). By integrating hypertriglyceridemia and hyperglycemia, the TyG index reflects both lipotoxic and glucotoxic metabolic stress. Hypertriglyceridemia has been linked to colorectal adenoma formation and metastatic potential in upper gastrointestinal malignancies (40), while chronic hyperglycemia has been associated with carcinogenesis through oxidative stress, mitochondrial dysfunction, and sustained inflammatory activation (41–45). Thus, TyG may represent a biologically meaningful composite indicator potentially linking metabolic stress to GI tumor development.

Beyond insulin and IGF-1 signaling, increasing evidence highlights the mechanistic importance of the PI3K/Akt/mTOR axis as a central integrator of nutrient sensing, energy metabolism, and cell proliferation in obesity-related carcinogenesis. The mammalian target of rapamycin (mTOR) functions as a nutrient-sensing kinase that coordinates anabolic metabolism, protein synthesis, and inflammatory signaling in response to hyperglycemia, hyperlipidemia, and hyperinsulinemia. Chronic activation of insulin signaling may stimulate PI3K/Akt-dependent mTOR activation, thereby promoting metabolic reprogramming and tumor cell growth in gastrointestinal tissues (46, 47). HFD exposure has been reported to enhance mTOR signaling activity, contributing to lipid metabolic dysregulation and inflammatory cytokine production (48, 49). Moreover, mTOR signaling interacts with inflammatory pathways and modulates the tumor microenvironment, thereby linking metabolic stress with immune dysregulation in cancer development (50). Systemic inflammation emerged as an important, though partial, mediator in this association. Mediation analyses identified the SII and NLR as significant mediators of the TyG–GI cancer relationship. SII integrates neutrophil, platelet, and lymphocyte counts, reflecting the balance between inflammatory activation and antitumor immune surveillance (8, 51–53). Elevated SII has been associated with a pro-inflammatory and immunosuppressed state that may be conducive to tumor initiation and progression. Similarly, NLR reflects the dominance of innate immune responses over adaptive immunity. Neutrophils have been suggested to promote tumor growth through cytokine secretion, DNA damage induction, activation of NF-κB signaling, and angiogenesis, while reduced lymphocyte counts may impair immune surveillance (54–57). Although the mediation proportions were modest (1.6% for SII and 2.8% for NLR), these findings suggest that inflammation may represent a biologically relevant pathway potentially linking insulin resistance to GI carcinogenesis, though the small effect sizes indicate that other pathways are likely to contribute substantially and warrant further investigation, consistent with the multifactorial nature of cancer development.

The animal experiments provided critical mechanistic support for the epidemiological findings. HFD–fed mice exhibited significant increases in body weight, triglycerides, total cholesterol, LDL-C, fasting blood glucose, and TyG, indicating pronounced metabolic dysregulation and systemic insulin resistance. Importantly, the elevation of TyG in the animal model closely mirrored the human observations, reinforcing the translational relevance of this marker. Histopathological analyses revealed dysplastic and precancerous alterations across multiple gastrointestinal organs, including the liver, colon, stomach, and pancreas. Moreover, elevated serum CA19-9 levels in high-fat diet–fed mice further suggest activation of tumorigenic pathways before overt malignancy becomes apparent (57, 58). Together, these findings provide experimental support for an association between HFD-related metabolic disturbances and early tumorigenic changes in the gastrointestinal tract.

This study demonstrates several important strengths. The TyG index showed superior predictive performance for GI cancer risk compared with conventional obesity-related indicators, and the identification of a threshold effect highlights the nonlinear relationship between metabolic dysfunction and cancer. Subgroup analyses further revealed significant heterogeneity in TyG-associated risk across sex and racial groups, emphasizing the need for individualized metabolic risk assessment. Notably, this study is the first to identify the SII and NLR as mediators linking insulin resistance–related metabolic stress to GI malignancies, reinforcing the central role of chronic inflammation in obesity-associated tumorigenesis. In addition, animal experiments provided mechanistic support by demonstrating that HFD–induced elevations in TyG are accompanied by pathological and tumorigenic alterations across multiple gastrointestinal organs. The use of NHANES data, with its stratified, multistage probability sampling design, further enhances the generalizability of these findings. From an expert perspective, the TyG index represents a low-cost, scalable, and clinically accessible biomarker with strong potential for real-world application. Unlike advanced imaging or molecular assays, TyG relies on routine laboratory measurements, facilitating integration into electronic health records and large-scale screening programs. Incorporating TyG into metabolic risk assessment frameworks may enable earlier identification of individuals at elevated GI cancer risk and support precision prevention strategies through dietary modification and metabolic intervention.

Nevertheless, several limitations should be acknowledged. First, the cross-sectional design of the NHANES dataset limits the ability to infer causal relationships between metabolic indicators and GI cancer risk. Given the cross-sectional nature of the data, the temporal sequence between metabolic dysfunction and cancer development remains uncertain, and reverse causation cannot be excluded, as cancer-related metabolic alterations may influence lipid and glucose parameters, thereby affecting TyG levels. Second, although major confounders—including age, sex, race, educational attainment, and family poverty–income ratio—were carefully adjusted for, the possibility of residual confounding from unmeasured or inadequately captured variables cannot be completely excluded. Although the complementary animal experiments provide biological plausibility for the observed associations, they do not establish direct causal equivalence in humans. Third, future longitudinal studies and mechanistic investigations in human populations are warranted to validate these findings and to further elucidate the biological pathways underlying the observed associations.

## 5 Conclusion

This study, based on a nationally representative NHANES dataset, provides evidence that HFD related metabolic dysregulation is associated with an elevated risk of GI cancers. A nonlinear, threshold-dependent association between the TyG and GI cancer risk was identified, highlighting TyG as a clinically meaningful biomarker. Systemic inflammation, as reflected by SII and NLR, partially mediates this association, underscoring the interplay between insulin resistance and chronic inflammatory activation. Importantly, animal experiments corroborated these findings by demonstrating that HFD-related elevation of TyG is associated with metabolic abnormalities and precancerous lesions across multiple GI organs. Collectively, these results advance the understanding of metabolic–inflammatory mechanisms underlying GI cancers and support the potential application of TyG-based strategies for early risk assessment, prevention, and intervention.

## Data Availability

Publicly available datasets were analyzed in this study. This data can be found at: the dataset(s) supporting the conclusions of this article is (are) available in the National Health and Nutrition Examination Survey (NHANES) (https://www.cdc.gov/nchs/nhanes/?CDC_AAref_Val=https://www.cdc.gov/nchs/nhanes/index.htm) and Global Burden of Disease Collaborative Network (GBD) (https://www.healthdata.org/research-analysis/gbd-data). The data supporting this article have been presented in the figures and tables (including Supplementary Figures and Tables). Raw data are available upon request from the authors.

## Footnotes

1
https://wwwn.cdc.gov/nchs/nhanes/analyticguidelines.aspx?utm_source

## Supplementary material

SUPPLEMENTARY FIGURE 1 Detailed questionnaire items.

SUPPLEMENTARY FIGURE 2 E-value analysis assessing the robustness of the association between TyG index and gastrointestinal cancer risk.

SUPPLEMENTARY FIGURE 3 Association between TyG and GI cancers. A solid rad line represents the smooth curve fit between variables. Blue bands represent the 95% of confidence interval from the fit. All adjusted for Age, sex, race, Family PIR, Education status, Married status.
