# Supplementary figures and images for: High-fat diet, triglyceride glucose index, and gastrointestinal cancer: integrative insights from human and animal studies
Source: Front Nutr. 2026 Apr 1;13:1734895. doi: 10.3389/fnut.2026.1734895 (PMC13079637; doi:10.3389/fnut.2026.1734895)

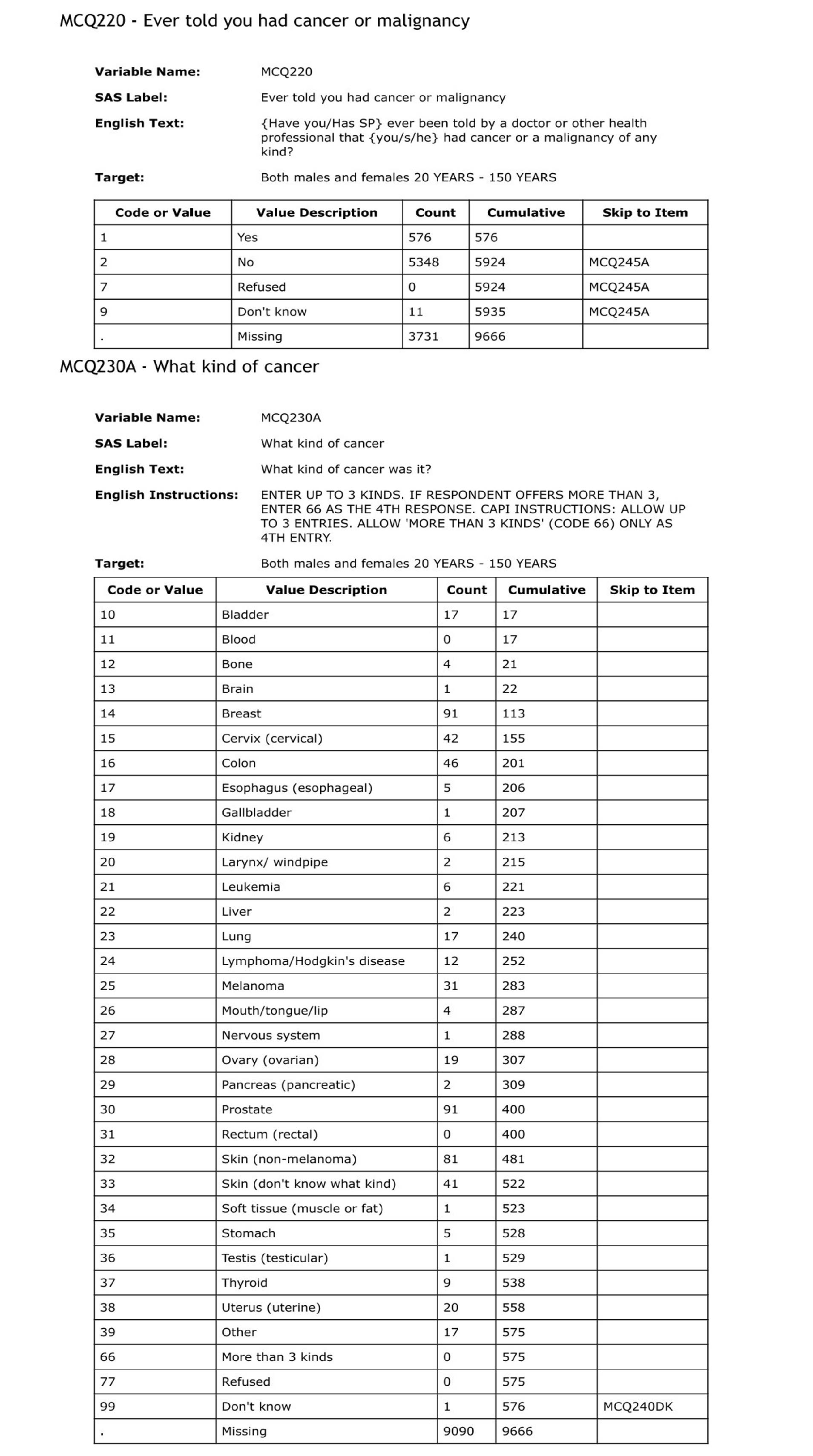

Supplement: SUPPLEMENTARY FIGURE 1 — Detailed questionnaire items. [file Image_1.jpg]

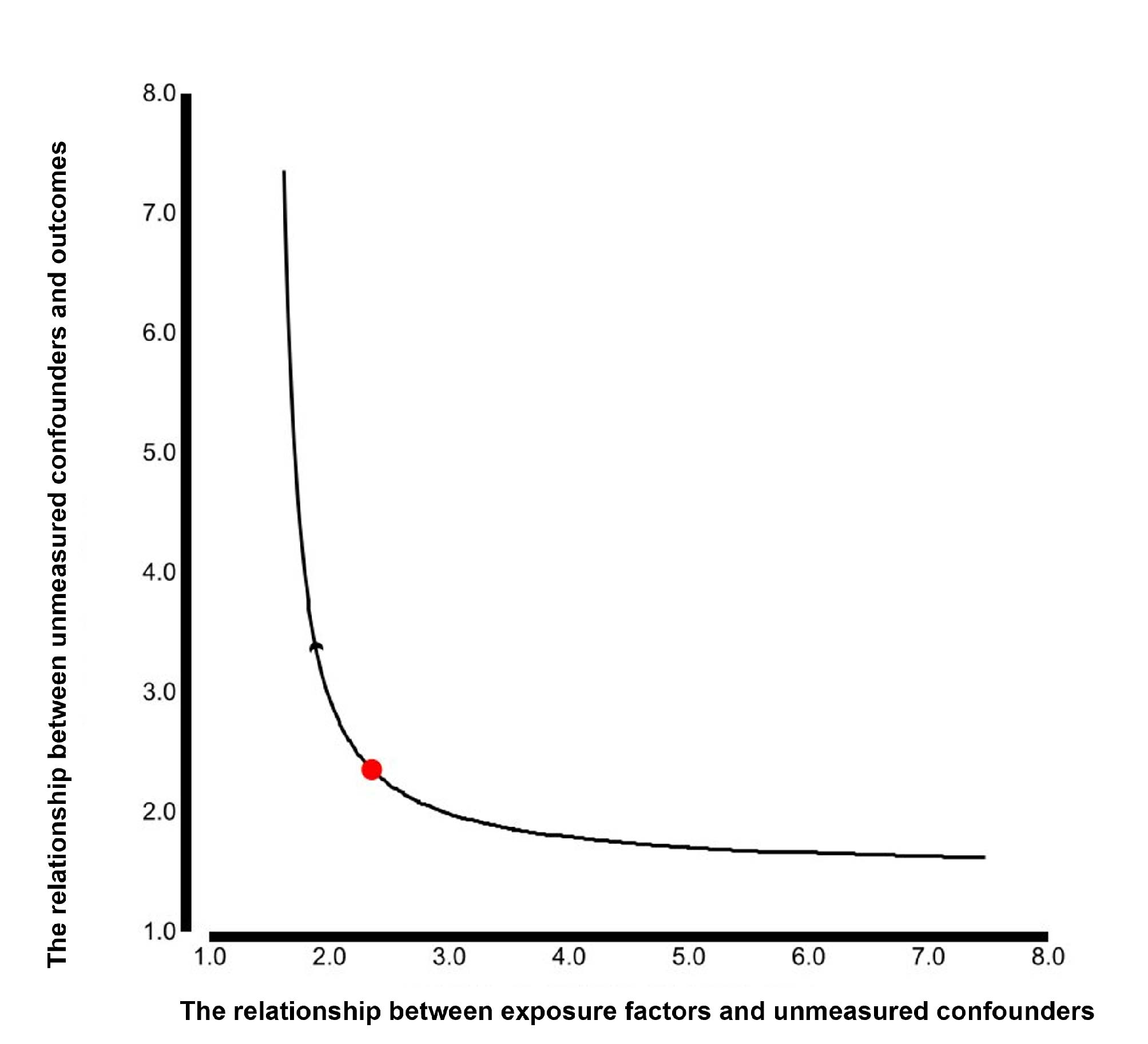

Supplement: SUPPLEMENTARY FIGURE 2 — E-value analysis assessing the robustness of the association between TyG index and gastrointestinal cancer risk. [file Image_2.jpg]

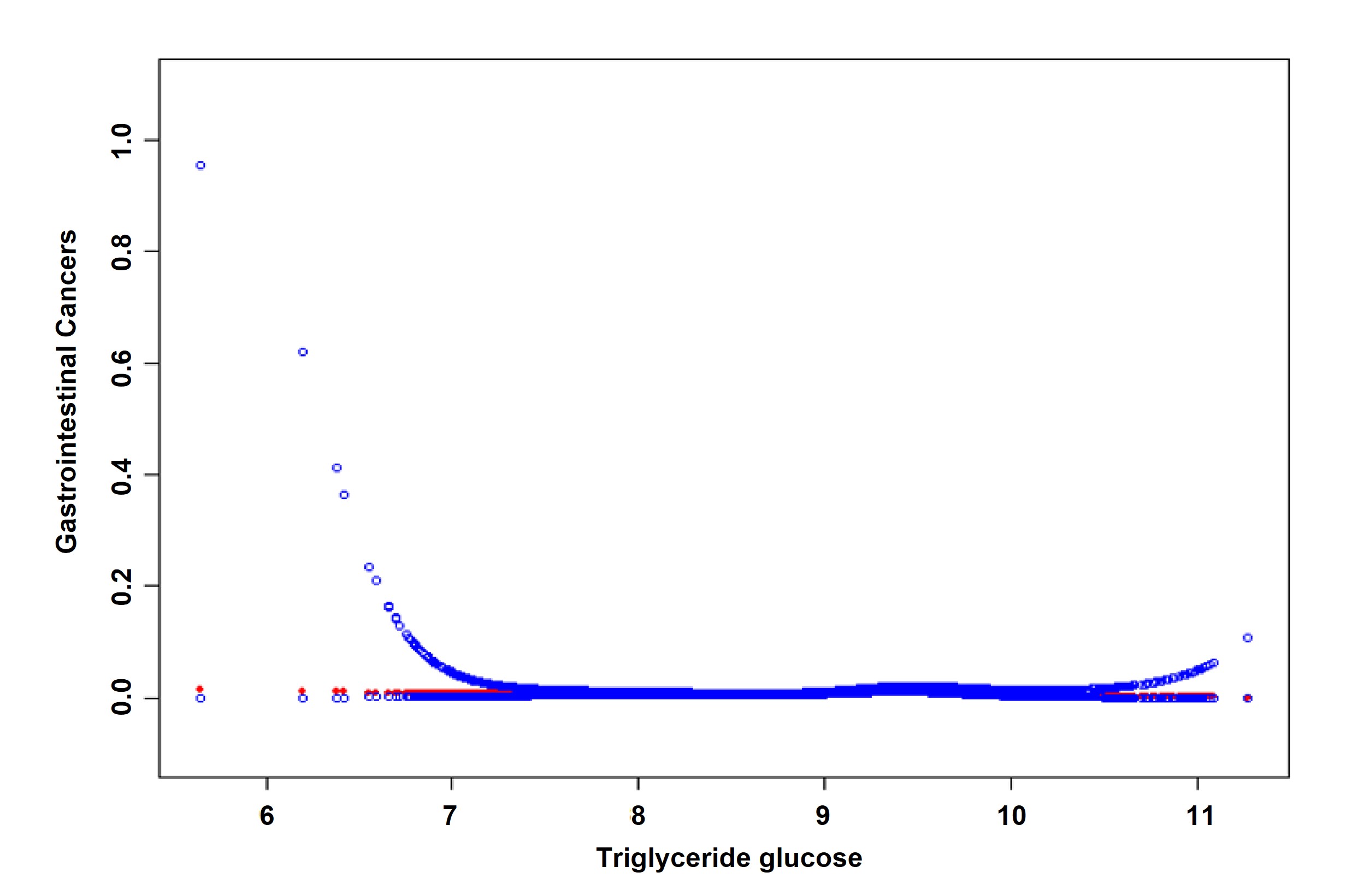

Supplement: SUPPLEMENTARY FIGURE 3 — Association between TyG and GI cancers. A solid rad line represents the smooth curve fit between variables. Blue bands represent the 95% of confidence interval from the fit. All adjusted for Age, sex, race, Family PIR, Education status, Married status. [file Image_3.jpg]
