# Supplementary material for: High-fat diet, triglyceride glucose index, and gastrointestinal cancer: integrative insights from human and animal studies
Source: Front Nutr. 2026 Apr 1;13:1734895. doi: 10.3389/fnut.2026.1734895 (PMC13079637; doi:10.3389/fnut.2026.1734895)
Supplement: Supplementary file 5 [file Table_2.docx]

Supplementary table 2: Subgroup analysis of the associations between lipid-related obesity indicators and GI cancer.

| Subgroup | OR (95%CI) , *P* value |
| --- | --- |
|  |  |
| Gender |  |
| Male | 1.015 (0.608, 1.695), 0.954 |
| Female | 2.148 (1.554, 2.969), <0.001 |
| Race/ethnicity |  |
| Mexican American | 0.999 (0.455, 2.194), 0.998 |
| Non-Hispanic | 1.594 (1.159, 2.192), 0.005 |
| Multiracial | 1.021 (0.441, 2.362), 0.961 |
